# {Bis[2-(diphenyl­phosphino)eth­yl]phenyl­phosphine-κ^3^ *P*,*P*′,*P*′′}chloridoplatinium(II) hexa­fluoridophosphate

**DOI:** 10.1107/S1600536809022405

**Published:** 2009-06-20

**Authors:** Scott A. Heston, Bruce C. Noll, Monte L. Helm

**Affiliations:** a Fort Lewis College, Department of Chemistry, 1000 Rim Drive, Durango, CO 81301, USA; b Bruker AXS, Inc., 5465 East Cheryl Parkway, Madison, WI 53711, USA

## Abstract

In the title compound, [PtCl(C_34_H_33_P_3_)]PF_6_, the Pt^II^ cation adopts a distorted square-planar PtClP_3_ geometry, arising from the *P*,*P*′,*P*′′-tridentate triphos ligand and a chloride ion. Four of the F atoms of the PF_6_^−^ anion are disordered over two sets of positions in a 0.614 (17):0.386 (17) ratio.

## Related literature

The corresponding complex with a Pd^II^ metal center is published concurently (Vorce *et al.*, 2009 ▶). The corresponding Pt^II^ complex has been previously reported as a CuCl_2_^−^ salt (Fernadez *et al., *2005). The corresponding complexes with both Pt^II^ and Pd^II^ have been previously reported as chloride and diphenyl­tetra­chlorido­stannate(IV) salts (Sevillano *et al.*, 1999*a* ▶; Garcia-Seijo *et al.*, 2001 ▶; Housecroft *et al.*, 1990 ▶). For other group 10–triphos complexes, see: Sevillano *et al.* (1999*b* ▶); Müller *et al.* (2000 ▶); Aizawa *et al.* (2002 ▶); Bertinsson *et al.* (1983 ▶); Autissier *et al.* (2005 ▶); Fernandez *et al.* (2005 ▶); King *et al.* (1971 ▶).
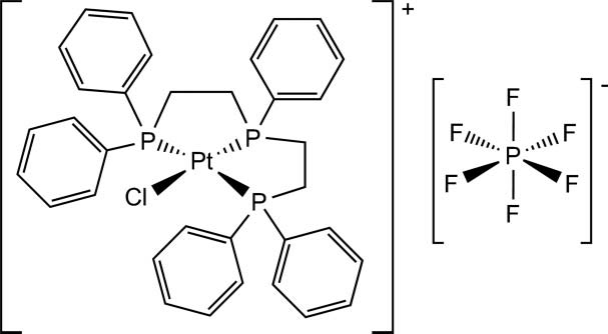


## Experimental

### 

#### Crystal data

[PtCl(C_34_H_33_P_3_)]PF_6_
*M* *_r_* = 910.02
Monoclinic, 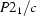

*a* = 11.3870 (11) Å
*b* = 19.6221 (18) Å
*c* = 16.4439 (16) Å
β = 107.528 (3)°
*V* = 3503.6 (6) Å^3^
*Z* = 4
Mo *K*α radiation
μ = 4.32 mm^−1^
*T* = 298 K
0.50 × 0.30 × 0.10 mm

#### Data collection

Bruker SMART X2S diffractometer
Absorption correction: multi-scan (*SADABS*; Sheldrick, 2008*a* ▶) *T*_min_ = 0.21, *T*_max_ = 0.65
21968 measured reflections
6155 independent reflections
5269 reflections with *I* > 2σ(*I*)
*R*_int_ = 0.025

#### Refinement

*R*[*F* ^2^ > 2σ(*F* ^2^)] = 0.020
*wR*(*F* ^2^) = 0.049
*S* = 1.03
6155 reflections
452 parameters
204 restraints
H-atom parameters constrained
Δρ_max_ = 0.58 e Å^−3^
Δρ_min_ = −0.41 e Å^−3^

### 

Data collection: *APEX2* (Bruker, 2008 ▶); cell refinement: *SAINT* (Bruker, 2008 ▶); data reduction: *SAINT* and *XPREP* (Bruker, 2008 ▶); program(s) used to solve structure: *SHELXS97* (Sheldrick, 2008*b* ▶); program(s) used to refine structure: *SHELXL97* (Sheldrick, 2008*b* ▶); molecular graphics: *SHELXTL* (Sheldrick, 2008*b* ▶); software used to prepare material for publication: *SHELXL97*.

## Supplementary Material

Crystal structure: contains datablocks global, I. DOI: 10.1107/S1600536809022405/hb2993sup1.cif

Structure factors: contains datablocks I. DOI: 10.1107/S1600536809022405/hb2993Isup2.hkl

Additional supplementary materials:  crystallographic information; 3D view; checkCIF report

**Fig. 1.**
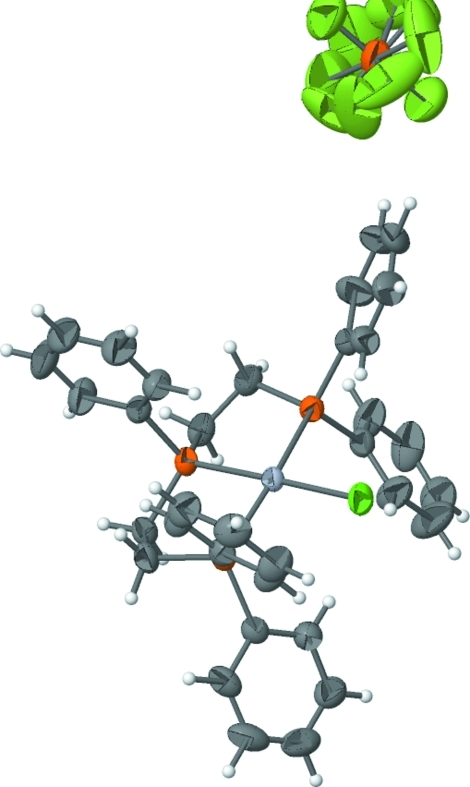
The molecular structure of (I), with 50% probability displacement ellipsoids.

Enhanced figure: interactive version of Fig. 1

## Figures and Tables

**Table 1:** Selected bond lengths (Å)

Pt1—P2	2.2095 (8)
Pt1—P3	2.3007 (8)
Pt1—P1	2.3185 (8)
Pt1—Cl1	2.3434 (8)

**Fig. 2.**
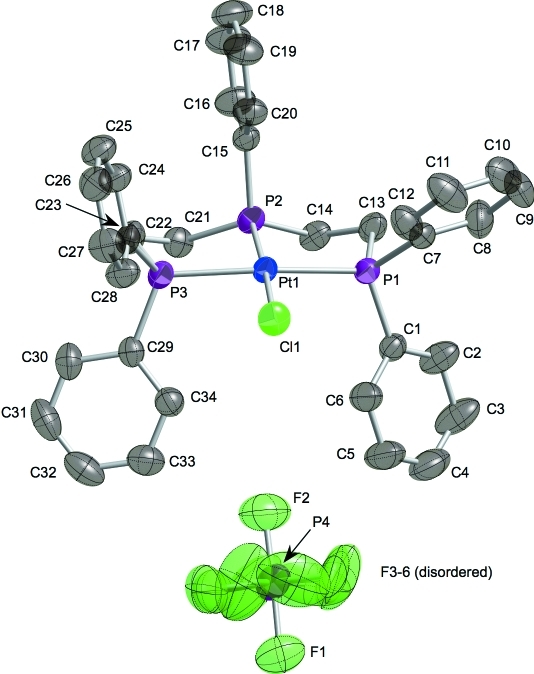
The molecular structure of (I), with atom labels and 50% probability displacement ellipsoids. Hydrogen atoms are omitted for clarity.

## supplementary crystallographic information

### Comment

The crystal structure of the title compound, (I), consists of a [Pt(triphos)Cl]^+^ cation and disordered PF_6_^-^ anion (Fig. 1). The cation shows a distorted square planar geometry (Table 1) around the metal center with a non-coordinating PF_6_^-^ anion.

### Experimental

The synthesis of (I) by a previously reported proceedure (King, *et al.*, 1971). Crystals where grown by slow solvent evaporation of a saturated dichloromethane solution of (I).

### Crystal data

**Table:**

[PtCl(C_34_H_33_P_3_)]PF_6_	*F*(000) = 1784
*M**_r_* = 910.02	*D*_x_ = 1.725 Mg m^−^^3^
Monoclinic, *P*2_1_/*c*	Mo *K*α radiation, λ = 0.71073 Å
Hall symbol: -P 2ybc	Cell parameters from 9957 reflections
*a* = 11.3870 (11) Å	θ = 2.5–25.1°
*b* = 19.6221 (18) Å	µ = 4.32 mm^−^^1^
*c* = 16.4439 (16) Å	*T* = 298 K
β = 107.528 (3)°	Plate, colorless
*V* = 3503.6 (6) Å^3^	0.50 × 0.30 × 0.10 mm
*Z* = 4	

### Data collection

**Table:**

Bruker SMART X2S diffractometer	6155 independent reflections
Radiation source: microfocus sealed tube, Bruker	5269 reflections with *I* > 2σ(*I*)
graphite	*R*_int_ = 0.025
Detector resolution: 8.33 pixels mm^-1^	θ_max_ = 25.1°, θ_min_ = 2.8°
ω scans	*h* = −13→13
Absorption correction: multi-scan (*SADABS*; Sheldrick, 2008a)	*k* = −18→23
*T*_min_ = 0.21, *T*_max_ = 0.65	*l* = −19→19
21968 measured reflections	

### Refinement

**Table:**

Refinement on *F*^2^	Primary atom site location: structure-invariant direct methods
Least-squares matrix: full	Secondary atom site location: difference Fourier map
*R*[*F*^2^ > 2σ(*F*^2^)] = 0.020	Hydrogen site location: inferred from neighbouring sites
*wR*(*F*^2^) = 0.049	H-atom parameters constrained
*S* = 1.03	*w* = 1/[σ^2^(*F*_o_^2^) + (0.0227*P*)^2^ + 1.641*P*] where *P* = (*F*_o_^2^ + 2*F*_c_^2^)/3
6155 reflections	(Δ/σ)_max_ = 0.002
452 parameters	Δρ_max_ = 0.58 e Å^−^^3^
204 restraints	Δρ_min_ = −0.41 e Å^−^^3^

### Special details

**Table:**

Geometry. All e.s.d.'s (except the e.s.d. in the dihedral angle between two l.s. planes) are estimated using the full covariance matrix. The cell e.s.d.'s are taken into account individually in the estimation of e.s.d.'s in distances, angles and torsion angles; correlations between e.s.d.'s in cell parameters are only used when they are defined by crystal symmetry. An approximate (isotropic) treatment of cell e.s.d.'s is used for estimating e.s.d.'s involving l.s. planes.
Refinement. The PF_6_ anion is disordered, showing alternate positions of the 4 *F* atoms F3—F6 when rotated about the axis F1—P4—F2. A second orientation for these equatorial positions was located. Based on the thermal parameters, additional positions are indicated, but were not modeled. To improve the quality of the fit for this anion, distance restraints (*SHELX* SADI) were added for the P–*F* bonds, as well as for the *F*–*F* interatomic distances. These account for 156 of the 204 restraints applied. The remaining restraints (ISOR) were applied to the anisotropic displacement parameters for the fluorine atoms.Refinement of *F*^2^ against ALL reflections. The weighted *R*-factor *wR* and goodness of fit *S* are based on *F*^2^, conventional *R*-factors *R* are based on *F*, with *F* set to zero for negative *F*^2^. The threshold expression of *F*^2^ > σ(*F*^2^) is used only for calculating *R*-factors(gt) *etc*. and is not relevant to the choice of reflections for refinement. *R*-factors based on *F*^2^ are statistically about twice as large as those based on *F*, and *R*- factors based on ALL data will be even larger.

### Fractional atomic coordinates and isotropic or equivalent isotropic displacement parameters (Å^2^)

**Table:**

	*x*	*y*	*z*	*U*_iso_*/*U*_eq_	Occ. (<1)
Pt1	0.139852 (10)	0.364806 (6)	0.374003 (7)	0.03173 (5)	
Cl1	0.31867 (8)	0.35123 (5)	0.48942 (5)	0.0498 (2)	
P1	0.22200 (7)	0.43573 (4)	0.29227 (5)	0.03695 (19)	
P2	−0.03648 (7)	0.37743 (4)	0.27189 (5)	0.03518 (19)	
P3	0.03774 (7)	0.28264 (4)	0.42630 (5)	0.03706 (19)	
C1	0.3286 (3)	0.39017 (18)	0.2487 (2)	0.0417 (8)	
C2	0.3950 (4)	0.3357 (2)	0.2925 (3)	0.0696 (12)	
H2	0.3851	0.3227	0.3444	0.084*	
C3	0.4758 (5)	0.3003 (3)	0.2602 (4)	0.0929 (17)	
H3	0.5209	0.2641	0.2909	0.111*	
C4	0.4896 (4)	0.3182 (3)	0.1838 (3)	0.0811 (14)	
H4	0.5422	0.2933	0.1614	0.097*	
C5	0.4268 (4)	0.3724 (3)	0.1397 (3)	0.0809 (15)	
H5	0.4382	0.3850	0.0881	0.097*	
C6	0.3458 (4)	0.4088 (2)	0.1716 (2)	0.0662 (12)	
H6	0.3030	0.4457	0.1413	0.079*	
C7	0.2923 (3)	0.51580 (17)	0.3367 (2)	0.0448 (8)	
C8	0.3091 (4)	0.5681 (2)	0.2843 (3)	0.0690 (12)	
H8	0.2889	0.5616	0.2257	0.083*	
C9	0.3563 (5)	0.6303 (2)	0.3202 (4)	0.0815 (15)	
H9	0.3673	0.6653	0.2852	0.098*	
C10	0.3863 (4)	0.6405 (2)	0.4054 (4)	0.0774 (15)	
H10	0.4162	0.6827	0.4283	0.093*	
C11	0.3726 (4)	0.5886 (2)	0.4579 (3)	0.0774 (14)	
H11	0.3950	0.5954	0.5166	0.093*	
C12	0.3255 (3)	0.5262 (2)	0.4237 (3)	0.0587 (10)	
H12	0.3162	0.4913	0.4595	0.070*	
C13	0.0924 (3)	0.46082 (19)	0.2000 (2)	0.0480 (9)	
H13A	0.0534	0.5012	0.2141	0.058*	
H13B	0.1226	0.4718	0.1524	0.058*	
C14	−0.0024 (3)	0.40301 (19)	0.1745 (2)	0.0459 (8)	
H14A	0.0311	0.3649	0.1510	0.055*	
H14B	−0.0766	0.4188	0.1320	0.055*	
C15	−0.1372 (3)	0.44123 (17)	0.2947 (2)	0.0392 (8)	
C16	−0.2526 (3)	0.4525 (2)	0.2376 (2)	0.0612 (11)	
H16	−0.2769	0.4292	0.1860	0.073*	
C17	−0.3320 (4)	0.4987 (2)	0.2575 (3)	0.0777 (13)	
H17	−0.4097	0.5061	0.2192	0.093*	
C18	−0.2964 (4)	0.5334 (2)	0.3334 (3)	0.0718 (12)	
H18	−0.3501	0.5642	0.3462	0.086*	
C19	−0.1830 (4)	0.5230 (2)	0.3901 (3)	0.0655 (11)	
H19	−0.1593	0.5468	0.4413	0.079*	
C20	−0.1031 (3)	0.47681 (18)	0.3711 (2)	0.0512 (9)	
H20	−0.0258	0.4696	0.4100	0.061*	
C21	−0.1212 (3)	0.29792 (17)	0.2618 (2)	0.0446 (8)	
H21A	−0.2052	0.3041	0.2255	0.054*	
H21B	−0.0824	0.2631	0.2368	0.054*	
C22	−0.1201 (3)	0.27699 (19)	0.3519 (2)	0.0500 (9)	
H22A	−0.1502	0.2307	0.3510	0.060*	
H22B	−0.1743	0.3067	0.3713	0.060*	
C23	0.0305 (3)	0.29942 (17)	0.5330 (2)	0.0413 (8)	
C24	−0.0603 (3)	0.3399 (2)	0.5480 (2)	0.0529 (9)	
H24	−0.1274	0.3533	0.5027	0.063*	
C25	−0.0518 (4)	0.3605 (2)	0.6305 (3)	0.0605 (11)	
H25	−0.1135	0.3874	0.6402	0.073*	
C26	0.0467 (4)	0.3415 (2)	0.6974 (2)	0.0590 (10)	
H26	0.0533	0.3564	0.7523	0.071*	
C27	0.1362 (4)	0.3003 (2)	0.6833 (2)	0.0614 (11)	
H27	0.2024	0.2865	0.7290	0.074*	
C28	0.1287 (3)	0.2791 (2)	0.6017 (2)	0.0538 (9)	
H28	0.1896	0.2512	0.5929	0.065*	
C29	0.1039 (3)	0.19806 (16)	0.42953 (19)	0.0387 (8)	
C30	0.0425 (4)	0.14216 (19)	0.4497 (2)	0.0539 (10)	
H30	−0.0322	0.1483	0.4609	0.065*	
C31	0.0923 (5)	0.0778 (2)	0.4532 (3)	0.0677 (12)	
H31	0.0515	0.0407	0.4673	0.081*	
C32	0.2015 (4)	0.0680 (2)	0.4359 (3)	0.0675 (12)	
H32	0.2343	0.0244	0.4381	0.081*	
C33	0.2626 (4)	0.1225 (2)	0.4153 (3)	0.0610 (11)	
H33	0.3362	0.1157	0.4029	0.073*	
C34	0.2149 (3)	0.18766 (18)	0.4131 (2)	0.0456 (8)	
H34	0.2575	0.2246	0.4005	0.055*	
P4	0.33951 (10)	0.86107 (6)	0.53542 (7)	0.0655 (3)	
F1	0.2344 (3)	0.91309 (17)	0.5407 (2)	0.1331 (13)	
F2	0.4424 (3)	0.81198 (16)	0.5283 (2)	0.1263 (12)	
F3	0.4311 (7)	0.8949 (6)	0.6137 (6)	0.170 (6)	0.614 (17)
F4	0.2458 (8)	0.8302 (5)	0.4551 (7)	0.181 (7)	0.614 (17)
F5	0.3788 (11)	0.9131 (4)	0.4771 (7)	0.143 (6)	0.614 (17)
F6	0.2992 (7)	0.8122 (5)	0.5916 (7)	0.196 (7)	0.614 (17)
F3'	0.3623 (16)	0.8459 (7)	0.6302 (5)	0.170 (11)	0.386 (17)
F4'	0.3028 (14)	0.8713 (10)	0.4399 (5)	0.184 (10)	0.386 (17)
F5'	0.4294 (9)	0.9199 (4)	0.5545 (15)	0.153 (11)	0.386 (17)
F6'	0.2434 (11)	0.8018 (5)	0.5150 (11)	0.141 (9)	0.386 (17)

### Atomic displacement parameters (Å^2^)

**Table:**

	*U*^11^	*U*^22^	*U*^33^	*U*^12^	*U*^13^	*U*^23^
Pt1	0.02685 (7)	0.03558 (8)	0.03213 (7)	−0.00116 (5)	0.00792 (5)	0.00172 (5)
Cl1	0.0352 (4)	0.0585 (6)	0.0467 (5)	−0.0016 (4)	−0.0013 (4)	0.0038 (4)
P1	0.0324 (4)	0.0385 (5)	0.0422 (5)	0.0009 (4)	0.0147 (4)	0.0056 (4)
P2	0.0290 (4)	0.0438 (5)	0.0311 (4)	0.0000 (4)	0.0066 (3)	−0.0002 (3)
P3	0.0322 (4)	0.0416 (5)	0.0385 (4)	−0.0023 (4)	0.0124 (4)	0.0045 (4)
C1	0.0343 (17)	0.046 (2)	0.0465 (19)	−0.0013 (15)	0.0144 (15)	−0.0008 (16)
C2	0.069 (3)	0.069 (3)	0.086 (3)	0.022 (2)	0.047 (3)	0.023 (2)
C3	0.095 (4)	0.080 (4)	0.126 (4)	0.042 (3)	0.067 (4)	0.029 (3)
C4	0.069 (3)	0.085 (4)	0.103 (4)	0.011 (3)	0.046 (3)	−0.021 (3)
C5	0.061 (3)	0.134 (5)	0.056 (3)	0.015 (3)	0.029 (2)	−0.004 (3)
C6	0.057 (2)	0.095 (3)	0.051 (2)	0.021 (2)	0.023 (2)	0.009 (2)
C7	0.0338 (18)	0.039 (2)	0.064 (2)	−0.0003 (15)	0.0188 (17)	0.0049 (17)
C8	0.081 (3)	0.051 (3)	0.087 (3)	−0.012 (2)	0.043 (3)	0.004 (2)
C9	0.083 (3)	0.046 (3)	0.122 (5)	−0.012 (2)	0.041 (3)	0.014 (3)
C10	0.047 (2)	0.049 (3)	0.122 (4)	−0.014 (2)	0.003 (3)	−0.006 (3)
C11	0.063 (3)	0.063 (3)	0.084 (3)	−0.006 (2)	−0.014 (2)	−0.005 (3)
C12	0.049 (2)	0.048 (2)	0.068 (3)	−0.0065 (18)	0.0007 (19)	0.0046 (19)
C13	0.0418 (19)	0.057 (2)	0.048 (2)	0.0080 (17)	0.0177 (16)	0.0167 (17)
C14	0.0408 (18)	0.063 (2)	0.0335 (17)	0.0071 (18)	0.0111 (14)	0.0050 (16)
C15	0.0340 (17)	0.042 (2)	0.0423 (18)	0.0025 (15)	0.0124 (15)	0.0026 (15)
C16	0.044 (2)	0.074 (3)	0.058 (2)	0.008 (2)	0.0042 (18)	−0.010 (2)
C17	0.045 (2)	0.084 (3)	0.094 (3)	0.020 (2)	0.006 (2)	−0.006 (3)
C18	0.057 (3)	0.064 (3)	0.098 (3)	0.015 (2)	0.028 (3)	−0.015 (3)
C19	0.066 (3)	0.063 (3)	0.067 (3)	0.008 (2)	0.019 (2)	−0.017 (2)
C20	0.045 (2)	0.052 (2)	0.053 (2)	0.0059 (17)	0.0090 (17)	−0.0046 (18)
C21	0.0377 (18)	0.044 (2)	0.0468 (19)	−0.0053 (15)	0.0043 (15)	−0.0068 (16)
C22	0.0346 (18)	0.055 (2)	0.058 (2)	−0.0089 (16)	0.0104 (16)	0.0066 (18)
C23	0.0398 (18)	0.045 (2)	0.0432 (18)	−0.0014 (15)	0.0189 (15)	0.0061 (15)
C24	0.047 (2)	0.063 (2)	0.051 (2)	0.0083 (19)	0.0199 (18)	0.0037 (19)
C25	0.060 (3)	0.069 (3)	0.062 (3)	0.009 (2)	0.032 (2)	0.000 (2)
C26	0.076 (3)	0.062 (3)	0.046 (2)	0.002 (2)	0.027 (2)	0.0018 (19)
C27	0.067 (3)	0.073 (3)	0.042 (2)	0.012 (2)	0.0129 (19)	0.0082 (19)
C28	0.055 (2)	0.062 (3)	0.046 (2)	0.0145 (19)	0.0190 (18)	0.0076 (18)
C29	0.0413 (18)	0.040 (2)	0.0339 (16)	−0.0049 (15)	0.0096 (14)	0.0008 (14)
C30	0.060 (2)	0.053 (3)	0.051 (2)	−0.0172 (19)	0.0205 (19)	−0.0012 (18)
C31	0.096 (4)	0.043 (3)	0.061 (3)	−0.020 (2)	0.020 (2)	0.0018 (19)
C32	0.086 (3)	0.042 (2)	0.063 (3)	0.008 (2)	0.005 (2)	−0.007 (2)
C33	0.057 (2)	0.053 (3)	0.069 (3)	0.007 (2)	0.012 (2)	−0.007 (2)
C34	0.045 (2)	0.043 (2)	0.049 (2)	−0.0006 (16)	0.0130 (16)	0.0008 (16)
P4	0.0518 (6)	0.0859 (9)	0.0608 (7)	0.0022 (6)	0.0199 (5)	0.0198 (6)
F1	0.085 (2)	0.135 (3)	0.193 (4)	0.031 (2)	0.062 (2)	0.027 (3)
F2	0.100 (2)	0.108 (3)	0.178 (3)	0.0275 (19)	0.052 (2)	−0.005 (2)
F3	0.082 (5)	0.309 (16)	0.115 (7)	−0.058 (7)	0.025 (4)	−0.069 (8)
F4	0.133 (9)	0.174 (11)	0.166 (11)	0.000 (7)	−0.061 (7)	−0.044 (8)
F5	0.200 (12)	0.121 (8)	0.155 (10)	0.039 (7)	0.122 (9)	0.062 (7)
F6	0.121 (6)	0.265 (13)	0.210 (13)	−0.036 (7)	0.061 (8)	0.170 (11)
F3'	0.27 (3)	0.174 (15)	0.051 (5)	0.124 (16)	0.023 (9)	0.000 (7)
F4'	0.183 (16)	0.31 (3)	0.047 (6)	−0.042 (14)	0.010 (8)	0.053 (10)
F5'	0.083 (8)	0.077 (7)	0.31 (3)	−0.036 (5)	0.079 (15)	−0.040 (13)
F6'	0.163 (15)	0.110 (10)	0.198 (19)	−0.074 (10)	0.130 (15)	−0.031 (10)

### Geometric parameters (Å, °)

**Table:**

Pt1—P2	2.2095 (8)	C17—C18	1.371 (6)
Pt1—P3	2.3007 (8)	C17—H17	0.9300
Pt1—P1	2.3185 (8)	C18—C19	1.361 (6)
Pt1—Cl1	2.3434 (8)	C18—H18	0.9300
P1—C7	1.814 (4)	C19—C20	1.384 (5)
P1—C1	1.819 (3)	C19—H19	0.9300
P1—C13	1.836 (3)	C20—H20	0.9300
P2—C15	1.812 (3)	C21—C22	1.535 (5)
P2—C21	1.815 (3)	C21—H21A	0.9700
P2—C14	1.828 (3)	C21—H21B	0.9700
P3—C23	1.812 (3)	C22—H22A	0.9700
P3—C29	1.817 (3)	C22—H22B	0.9700
P3—C22	1.848 (3)	C23—C24	1.383 (5)
C1—C2	1.380 (5)	C23—C28	1.387 (5)
C1—C6	1.389 (5)	C24—C25	1.392 (5)
C2—C3	1.381 (6)	C24—H24	0.9300
C2—H2	0.9300	C25—C26	1.365 (6)
C3—C4	1.359 (6)	C25—H25	0.9300
C3—H3	0.9300	C26—C27	1.375 (5)
C4—C5	1.362 (6)	C26—H26	0.9300
C4—H4	0.9300	C27—C28	1.382 (5)
C5—C6	1.388 (6)	C27—H27	0.9300
C5—H5	0.9300	C28—H28	0.9300
C6—H6	0.9300	C29—C34	1.385 (4)
C7—C12	1.381 (5)	C29—C30	1.393 (5)
C7—C8	1.390 (5)	C30—C31	1.379 (5)
C8—C9	1.390 (6)	C30—H30	0.9300
C8—H8	0.9300	C31—C32	1.370 (6)
C9—C10	1.352 (7)	C31—H31	0.9300
C9—H9	0.9300	C32—C33	1.372 (6)
C10—C11	1.375 (6)	C32—H32	0.9300
C10—H10	0.9300	C33—C34	1.385 (5)
C11—C12	1.386 (5)	C33—H33	0.9300
C11—H11	0.9300	C34—H34	0.9300
C12—H12	0.9300	P4—F6	1.496 (5)
C13—C14	1.535 (5)	P4—F5'	1.512 (8)
C13—H13A	0.9700	P4—F4'	1.512 (8)
C13—H13B	0.9700	P4—F3'	1.530 (8)
C14—H14A	0.9700	P4—F3	1.542 (7)
C14—H14B	0.9700	P4—F2	1.548 (3)
C15—C16	1.383 (5)	P4—F4	1.549 (6)
C15—C20	1.387 (5)	P4—F5	1.556 (6)
C16—C17	1.387 (5)	P4—F6'	1.563 (8)
C16—H16	0.9300	P4—F1	1.595 (3)
			
P2—Pt1—P3	85.22 (3)	C18—C19—C20	119.7 (4)
P2—Pt1—P1	85.71 (3)	C18—C19—H19	120.1
P3—Pt1—P1	167.13 (3)	C20—C19—H19	120.1
P2—Pt1—Cl1	175.83 (3)	C19—C20—C15	120.7 (3)
P3—Pt1—Cl1	91.79 (3)	C19—C20—H20	119.7
P1—Pt1—Cl1	97.69 (3)	C15—C20—H20	119.7
C7—P1—C1	108.56 (15)	C22—C21—P2	107.0 (2)
C7—P1—C13	104.36 (16)	C22—C21—H21A	110.3
C1—P1—C13	105.78 (16)	P2—C21—H21A	110.3
C7—P1—Pt1	119.51 (12)	C22—C21—H21B	110.3
C1—P1—Pt1	111.53 (12)	P2—C21—H21B	110.3
C13—P1—Pt1	105.99 (11)	H21A—C21—H21B	108.6
C15—P2—C21	105.12 (16)	C21—C22—P3	110.3 (2)
C15—P2—C14	107.82 (16)	C21—C22—H22A	109.6
C21—P2—C14	113.75 (16)	P3—C22—H22A	109.6
C15—P2—Pt1	114.03 (11)	C21—C22—H22B	109.6
C21—P2—Pt1	108.04 (11)	P3—C22—H22B	109.6
C14—P2—Pt1	108.22 (11)	H22A—C22—H22B	108.1
C23—P3—C29	106.08 (15)	C24—C23—C28	118.9 (3)
C23—P3—C22	109.46 (16)	C24—C23—P3	122.1 (3)
C29—P3—C22	106.05 (16)	C28—C23—P3	118.4 (3)
C23—P3—Pt1	114.22 (11)	C23—C24—C25	120.3 (4)
C29—P3—Pt1	113.54 (11)	C23—C24—H24	119.9
C22—P3—Pt1	107.16 (11)	C25—C24—H24	119.9
C2—C1—C6	118.3 (3)	C26—C25—C24	120.4 (4)
C2—C1—P1	120.0 (3)	C26—C25—H25	119.8
C6—C1—P1	121.6 (3)	C24—C25—H25	119.8
C1—C2—C3	120.9 (4)	C25—C26—C27	119.7 (4)
C1—C2—H2	119.6	C25—C26—H26	120.1
C3—C2—H2	119.6	C27—C26—H26	120.1
C4—C3—C2	120.1 (4)	C26—C27—C28	120.6 (4)
C4—C3—H3	120.0	C26—C27—H27	119.7
C2—C3—H3	120.0	C28—C27—H27	119.7
C3—C4—C5	120.4 (4)	C27—C28—C23	120.2 (3)
C3—C4—H4	119.8	C27—C28—H28	119.9
C5—C4—H4	119.8	C23—C28—H28	119.9
C4—C5—C6	120.2 (4)	C34—C29—C30	118.9 (3)
C4—C5—H5	119.9	C34—C29—P3	121.6 (3)
C6—C5—H5	119.9	C30—C29—P3	119.5 (3)
C5—C6—C1	120.1 (4)	C31—C30—C29	120.1 (4)
C5—C6—H6	119.9	C31—C30—H30	120.0
C1—C6—H6	119.9	C29—C30—H30	120.0
C12—C7—C8	119.1 (4)	C32—C31—C30	120.5 (4)
C12—C7—P1	119.7 (3)	C32—C31—H31	119.8
C8—C7—P1	121.1 (3)	C30—C31—H31	119.8
C7—C8—C9	119.5 (4)	C31—C32—C33	120.1 (4)
C7—C8—H8	120.3	C31—C32—H32	119.9
C9—C8—H8	120.3	C33—C32—H32	119.9
C10—C9—C8	121.0 (4)	C32—C33—C34	120.1 (4)
C10—C9—H9	119.5	C32—C33—H33	119.9
C8—C9—H9	119.5	C34—C33—H33	119.9
C9—C10—C11	119.9 (4)	C29—C34—C33	120.3 (3)
C9—C10—H10	120.0	C29—C34—H34	119.9
C11—C10—H10	120.0	C33—C34—H34	119.9
C10—C11—C12	120.2 (5)	F5'—P4—F4'	94.2 (8)
C10—C11—H11	119.9	F5'—P4—F3'	92.3 (7)
C12—C11—H11	119.9	F4'—P4—F3'	173.1 (8)
C7—C12—C11	120.2 (4)	F6—P4—F3	90.9 (5)
C7—C12—H12	119.9	F6—P4—F2	91.6 (4)
C11—C12—H12	119.9	F5'—P4—F2	90.6 (4)
C14—C13—P1	110.6 (2)	F4'—P4—F2	89.0 (5)
C14—C13—H13A	109.5	F3'—P4—F2	93.2 (4)
P1—C13—H13A	109.5	F3—P4—F2	89.6 (4)
C14—C13—H13B	109.5	F6—P4—F4	90.9 (5)
P1—C13—H13B	109.5	F3—P4—F4	177.5 (5)
H13A—C13—H13B	108.1	F2—P4—F4	92.0 (3)
C13—C14—P2	106.3 (2)	F6—P4—F5	178.6 (5)
C13—C14—H14A	110.5	F3—P4—F5	89.1 (4)
P2—C14—H14A	110.5	F2—P4—F5	89.8 (3)
C13—C14—H14B	110.5	F4—P4—F5	89.0 (5)
P2—C14—H14B	110.5	F5'—P4—F6'	178.3 (6)
H14A—C14—H14B	108.7	F4'—P4—F6'	85.3 (8)
C16—C15—C20	118.9 (3)	F3'—P4—F6'	88.1 (6)
C16—C15—P2	120.1 (3)	F2—P4—F6'	91.0 (4)
C20—C15—P2	120.9 (3)	F6—P4—F1	90.1 (4)
C15—C16—C17	119.9 (4)	F5'—P4—F1	88.4 (4)
C15—C16—H16	120.1	F4'—P4—F1	89.7 (5)
C17—C16—H16	120.1	F3'—P4—F1	88.2 (4)
C18—C17—C16	120.2 (4)	F3—P4—F1	90.5 (4)
C18—C17—H17	119.9	F2—P4—F1	178.3 (2)
C16—C17—H17	119.9	F4—P4—F1	87.8 (3)
C19—C18—C17	120.6 (4)	F5—P4—F1	88.5 (3)
C19—C18—H18	119.7	F6'—P4—F1	90.0 (4)
C17—C18—H18	119.7		
